# Tethering Sister Centromeres to Each Other Suggests the Spindle Checkpoint Detects Stretch within the Kinetochore

**DOI:** 10.1371/journal.pgen.1004492

**Published:** 2014-08-07

**Authors:** Natalie J. Nannas, Andrew W. Murray

**Affiliations:** 1 Molecular and Cellular Biology Department, Harvard University, Cambridge, Massachusetts, United States of America; 2 FAS Center for Systems Biology, Harvard University, Cambridge, Massachusetts, United States of America; Stowers Institute for Medical Research, United States of America

## Abstract

The spindle checkpoint ensures that newly born cells receive one copy of each chromosome by preventing chromosomes from segregating until they are all correctly attached to the spindle. The checkpoint monitors tension to distinguish between correctly aligned chromosomes and those with both sisters attached to the same spindle pole. Tension arises when sister kinetochores attach to and are pulled toward opposite poles, stretching the chromatin around centromeres and elongating kinetochores. We distinguished between two hypotheses for where the checkpoint monitors tension: between the kinetochores, by detecting alterations in the distance between them, or by responding to changes in the structure of the kinetochore itself. To distinguish these models, we inhibited chromatin stretch by tethering sister chromatids together by binding a tetrameric form of the Lac repressor to arrays of the Lac operator located on either side of a centromere. Inhibiting chromatin stretch did not activate the spindle checkpoint; these cells entered anaphase at the same time as control cells that express a dimeric version of the Lac repressor, which cannot cross link chromatids, and cells whose checkpoint has been inactivated. There is no dominant checkpoint inhibition when sister kinetochores are held together: cells expressing the tetrameric Lac repressor still arrest in response to microtubule-depolymerizing drugs. Tethering chromatids together does not disrupt kinetochore function; chromosomes are successfully segregated to opposite poles of the spindle. Our results indicate that the spindle checkpoint does not monitor inter-kinetochore separation, thus supporting the hypothesis that tension is measured within the kinetochore.

## Author Summary

The spindle checkpoint monitors tension on chromosomes to distinguish between chromosomes that are correctly and incorrectly attached to the spindle. Tension is generated across a correctly attached chromosome as microtubules from opposite poles attach to and pull kinetochores apart, but are resisted by the cohesin that holds sister chromatids together. This tension generates separation between kinetochores as pericentric chromatin stretches and it also elongates the kinetochores. To monitor tension, the checkpoint could measure the separation between kinetochores or the stretch within them. We inhibited the ability of pericentric chromatin to stretch by tethering sister centromeres to each other, and we asked whether the resulting reduction in inter-kinetochore separation artificially activated the spindle checkpoint. Inhibiting inter-kinetochore separation does not delay anaphase, and the timing of mitosis was the same in cells with or without the spindle checkpoint, showing that the checkpoint is not activated. Inhibiting chromatin stretch does not alter the function of kinetochores as chromosomes are still segregated correctly, nor does it hinder the checkpoint. Cells whose sister kinetochores are held together can still activate the checkpoint in response to microtubule depolymerization. Our results indicate the spindle checkpoint does not monitor inter-kinetochore separation and likely monitors tension within kinetochores.

## Introduction

Faithful chromosome segregation is essential. Mistakes lead to aneuploidy [1], cancer progression [2], and birth defects [3]. To ensure proper division of chromosomes, eukaryotes have evolved the spindle checkpoint, which monitors the kinetochore, a large multi-protein complex that assembles on centromeric DNA and attaches microtubules to chromosomes. In *Saccharomyces cerevisiae*, the budding yeast, the kinetochore consists of over 65 proteins that are assembled on the conserved 125 bp centromere [4]. The spindle checkpoint delays the onset of chromosome segregation until all chromosomes have attached their two sister kinetochores to microtubules emanating from opposite poles (bi-orientation) [5], [6]; it is activated by unattached kinetochores [5], [7] and lack of tension at the kinetochore [8], [9]. Morphologically, the checkpoint regulates the transition between metaphase, when the pairs of sister chromatids are aligned equidistant from the two poles, and anaphase, when the sisters split apart and are pulled to opposite poles.

Bi-oriented kinetochores are under tension: microtubules pull them towards the poles, but the chromosomes they lie on are held together by cohesin. In metaphase, this tension can be seen as separation of GFP-labeled centromeres [10], [11] and by elongation of the kinetochores, detected by measuring the separation between different kinetochore proteins [12]–[14]. In budding yeast, removing tension (by preventing replication or uncoupling sister chromatids) activates the spindle checkpoint and arrests cells in mitosis [9]. An unpaired, tensionless chromosome in praying mantid spermatocytes delays cell division, and applying tension to this chromosome allows cells to enter anaphase [8]. Although there is debate about whether the release of tension, or the subsequent release of microtubules from the kinetochore, generates the molecular signal that arrests cells in mitosis, it is clear that kinetochores can monitor tension, thus controlling the stability of microtubule attachment and progress through mitosis. The release of chromosomes and subsequent cell cycle arrest by the spindle checkpoint requires the activity of Sgo1 and the protein kinase, Ipl1/Aurora B [15]–[19].

**Figure 1:**
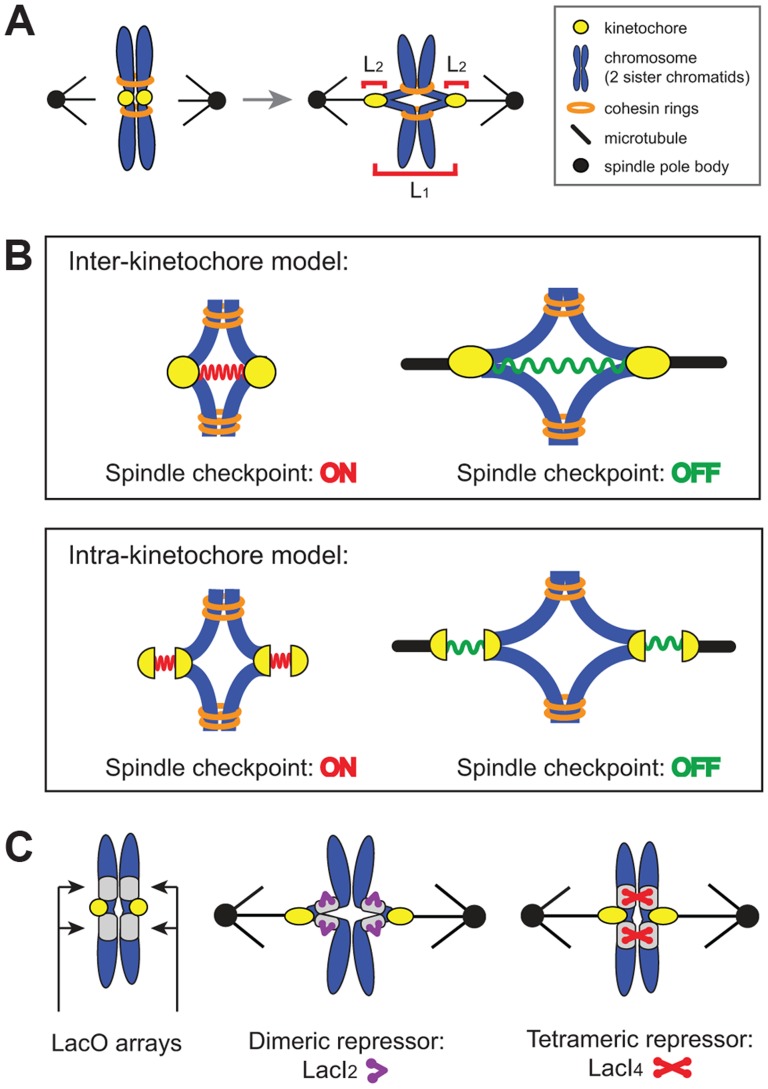
The spindle checkpoint is sensitive to tension on bi-oriented chromosomes. (**A**) Unattached or incorrectly attached chromosomes produce no tension; correct attachments (bi-orientation) occur when sister chromatids (blue) attach to opposite poles (black dot) via kinetochores (yellow dot). Tension is generated as microtubules attempt to pull chromatids apart but are resisted by cohesin (orange rings). Bi-orientation tension creates separation between kinetochores (L_1_) and separation within kinetochores (L_2_). (**B**) The spindle checkpoint monitors tension either between kinetochores (inter-kinetochore models) or within kinetochores (intra-kinetochore models). (**C**) To distinguish between models, pericentric chromatin stretching (inter-kinetochore distance) was inhibited by tethering chromatids together with the cross-linking properties of the Lac repressor. Lac operator arrays (gray boxes) are placed on either side of the centromere and either a dimeric (purple) or tetrameric repressor (red) is expressed. The dimeric form of the repressor contains a C-terminal truncation that prevents tethering, while the tetrameric form can crosslink two chromatids.

Where does the spindle checkpoint measure tension? There are two possible locations: between the two sister kinetochores (inter-kinetochore, L_1_ in Figure 1A) or within an individual kinetochore (intra-kinetochore, L_2_ in Figure 1A). Inter-kinetochore tension could be measured by the stretching of pericentric chromatin [11], or by a protein spring that spans the distance between kinetochores, such as PICH [20], a protein seen to span the inter-kinetochore gap in HeLa cells [21], [22] (Figure 1B). Intra-kinetochore stretch could be detected by monitoring changes in the distance between different parts of the kinetochore or conformational change in a single molecule. For either model, stretch stabilizes microtubule attachment to the kinetochore and relaxation destabilizes attachment and activates the checkpoint [12], [13], [23] (Figure 1B).

We manipulated budding yeast chromosomes to determine whether inter- or intra-kinetochore stretch regulates the spindle checkpoint (Figure 1C). By binding the tetrameric form of the GFP-labeled Lac repressor to an array of Lac operators, we held sister centromeres together (and measured their separation), inhibiting inter-kinetochore separation as cells entered mitosis. Despite the inhibited inter-kinetochore stretch, sister chromatids still separated on schedule, even though our manipulation left cells capable of assembling functional kinetochores and activating the spindle checkpoint. Because inhibiting inter-kinetochore separation does not slow mitosis, we believe that the spindle checkpoint senses tension by monitoring events within the kinetochore.

## Results

### Tethering chromatids together with tetrameric Lac repressor inhibits chromatin stretch

**Figure 2:**
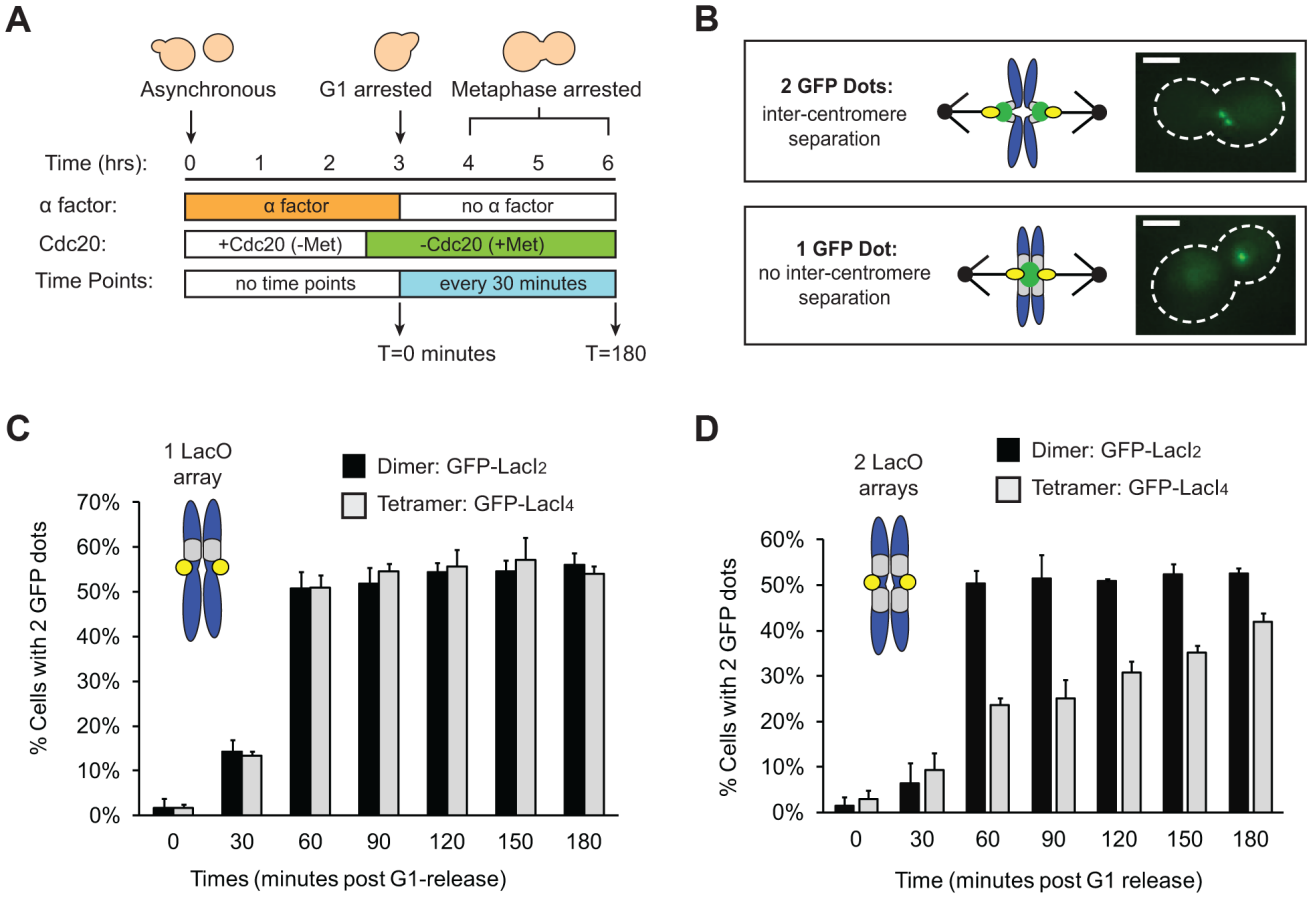
Tetrameric Lac repressor inhibits sister chromatid stretching. (**A**) To measure the stretching of chromatids, asynchronous populations were treated with alpha factor to arrest cells in G1. Cells were released from G1 into a metaphase arrest generated by depletion of Cdc20, an essential co-activator of the Anaphase Promoting Complex; samples were taken every 30 minutes and scored for separated chromatids (n>100). (**B**) Both versions of the repressor are fused to GFP to visualize sister chromatids. Separated chromatids appear as two GFP dots, and one GFP dot is categorized as no separation. Scale bar is 3 µm. (**C**) Inter-kinetochore separation is not inhibited if a LacO array is placed on only one side of the centromere. Both control, dimeric repressor (GFP-LacI_2_) cells and tetrameric repressor (GFP-LacI_4_) cells reach maximum percent separation 60 minutes after release from G1; there is no statistical difference between dimer and tetramer cells at all time points. (**D**) Centromere separation is inhibited if the tetrameric form of the Lac repressor (GFP-LacI_4_) is expressed and LacO arrays are placed on both sides of the centromere. Control cells expressing the dimeric repressor (GFP-LacI_2_) reach maximum stretching 60 minutes post-release from G1; the tetrameric strain has fewer cells with visibly stretched chromatids at all time points in metaphase arrest (*p*<0.005, Student's *t*-test). More than 100 cells were scored for GFP dots for each strain in each experiment; error bars represent standard deviation across 3 independent trials.

Tension on bi-oriented chromosomes allows the spindle checkpoint to distinguish between correct and incorrect attachments. Tension increases the separation between the centromeres and the kinetochores that have been assembled on them [10], [11] (L_1_ in Figure 1A) and the separation between components in a single kinetochore [12]–[14] (L_2_ in Figure 1A), but we do not know which distance the checkpoint monitors. To reduce the inter-kinetochore distance (L_1_), we tethered the sister chromatids of Chromosome III to each other by placing Lac operator (LacO) arrays on either side of the centromere and expressing two alternative versions of the Lac repressor. The tetrameric Lac repressor (LacI_4_) can bind simultaneously to two chromatids thus holding them together. The dimeric form of the repressor (LacI_2_) [24] is a control; it binds the Lac operator, but the two DNA binding domains must bind to the same operator, preventing the dimer from holding two DNA molecules together. It has been previously demonstrated that the tetrameric Lac repressor can hold homologous sister chromosomes together during meiosis in budding yeast while the dimeric Lac repressor cannot [25]. Both repressors were fused to GFP to see the centromeric DNA. Centromeric separation gives rise to two GFP dots [10], [11], and one GFP dot indicates two centromeres separated by less than the resolution of light microscopy, which is theoretically 200 nm, but is probably closer to 350 nm in our hands (Figure 2B). Both repressors contained two point mutations (P3Y and S61L) in the DNA-binding domain to produce the tightest binding affinity of all characterized Lac repressors (*K_d_*≈10^−15^ M) [26].

We asked if the tetrameric Lac repressor inhibits centromere separation in metaphase. Cells were synchronized in G1 by treating them with the mating pheromone, alpha factor, released from this arrest, and allowed to proceed to a metaphase arrest, caused by removal of Cdc20, an essential activator of the anaphase promoting complex (APC, Figure 2A). Cells expressing GFP-LacI_2_ or GFP-LacI_4_ were sampled every 30 minutes for 3 hours and examined by fluorescence microscopy. Their centromeres were scored as stretched apart (2 GFP dots) or unstretched (1 GFP dot) (Figure 2B). We initially placed a Lac operator array on only one side of the centromere, but we found that a single array did not inhibit the separation of the centromeres (Figure 2C). Both dimer- and tetramer-expressing cells containing an array on one side of the centromere had equivalent percentage of visibly separated centromeres at all time points; there was no statistical difference between the two populations (*p*>0.35 at all time points, Student's *t*-test).

To better tether the two chromatids together, we placed Lac operator arrays on both sides of the centromere (Figure 2D). For the first 30 minutes after their release from alpha factor, control (GFP-LacI_2_) or tethered (GFP-LacI_4_) cells both showed little centromere separation (<10% stretched) consistent with cells being in S phase and lacking a spindle. At 60 minutes, cells were entering mitosis: 50±3% of control, GFP-LacI_2_ cells (n>100) had 2 GFP dots whereas only 24±2% of tethered GFP-LacI_4_ cells (n>100) had 2 dots (*p*<0.005, Student's *t*-test, Figure 2D). Throughout the remaining time points, approximately 50% of control GFP-LacI_2_ cells had 2 dots, similar to previous studies [10], [11]. Cells expressing GFP-LacI_4_ had significantly lower percentage of visible inter-kinetochore separation at all time points (*p*<0.005, Student's *t*-test), but the fraction rose during the metaphase arrest from 24±2% at 60 minutes to 42±2% at 180 minutes (*p*<0.005, Student's *t*-test). This experiment shows that the tetrameric Lac repressor can reduce inter-kinetochore separation only if Lac operator arrays are placed on both sides of the centromere, and reveals that this effect is primarily kinetic: the fraction of cells with visibly separated centromeres rises slowly during a prolonged metaphase arrest.

**Figure 3:**
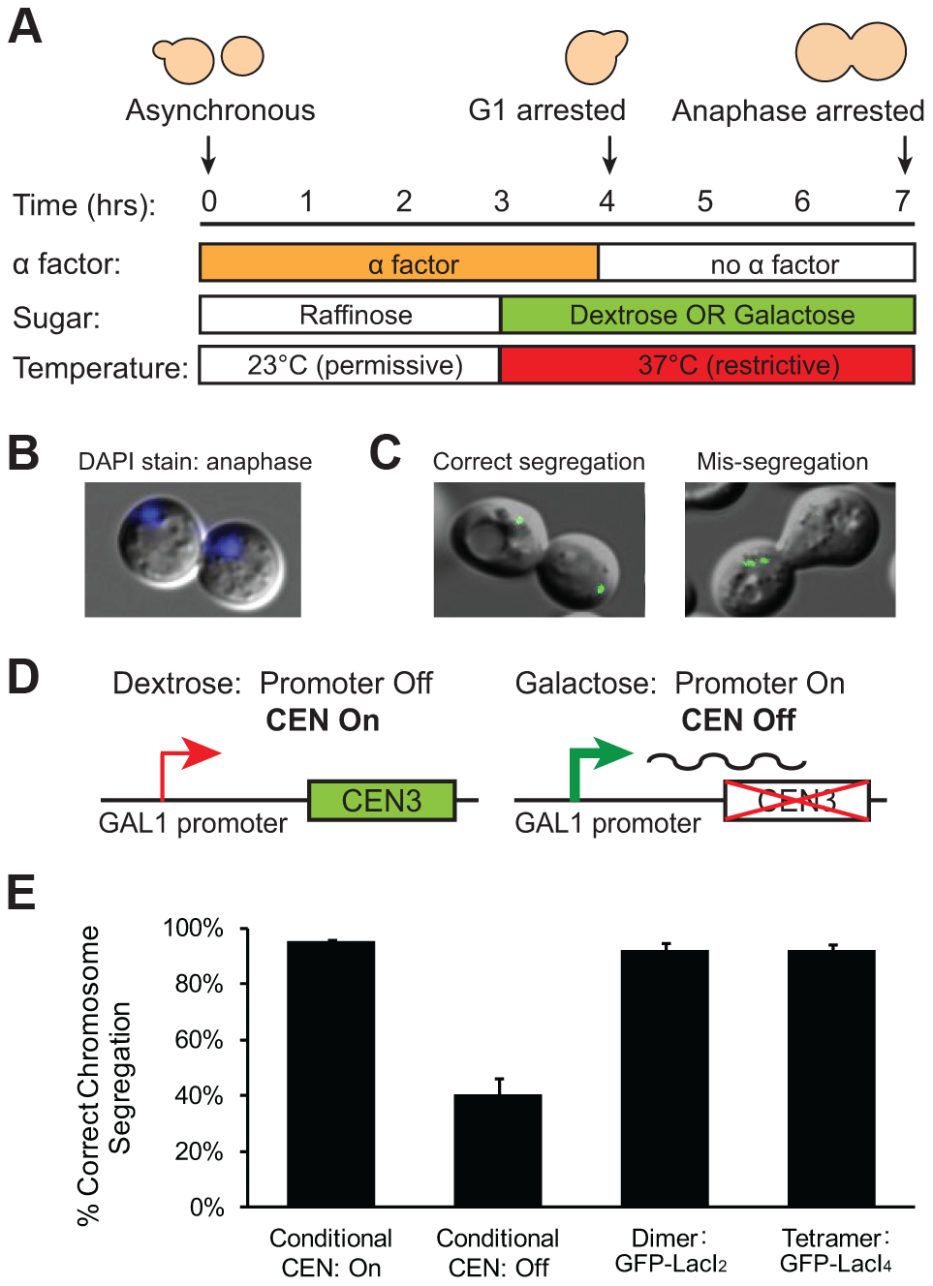
Holding sister centromeres close together does not disrupt chromosome segregation. (**A**) To determine if the tetrameric Lac repressor disrupts kinetochore assembly or error correction mechanisms, cells were grown in YP +2% raffinose at the permissive temperature and synchronized in G1 with alpha factor. Cells were washed and released into an anaphase arrest at the restrictive temperature in either YP +2% glucose or +2% galactose; anaphase arrest was induced by the *cdc15-2* temperature sensitive allele. The segregation of GFP-labeled chromatids was assessed three hours after release from G1. (**B**) DAPI staining was used to confirm anaphase arrest in *cdc15-2* cells grown at the restrictive temperature. Anaphase cells contain a DNA mass in both the mother and daughter cell; 99±1.5% of scored cells had a DAPI staining pattern corresponding to anaphase. (**C**) Correctly segregated chromosomes were scored as cells in which both the mother and daughter cell received a copy of the chromosome (one GFP dot in each cell). Chromosome mis-segregation was scored as cells in which both copies of the chromosome were located in one cell. (**D**) A conditional centromere was used as a positive control for chromosome mis-segregation. When grown in glucose, the *GAL1* promoter placed upstream of *CEN3* is turned off and the centromere is functional; however, when grown in galactose, the *GAL1* promoter is turned on and the centromere is disrupted by the transcriptional machinery. (**E**) Cells expressing the tetrameric repressor do not have increased rates of chromosome mis-segregation compared to those expressing the dimeric repressor or cells with their conditional centromere turned on. However, there was a large and statistically significant difference between these three strains and cells with their conditional centromeres turned off, which were as likely to mis-segregate sister chromatids as they were to segregate them properly. 200 cells scored for correct chromosome segregation for each strain in each experiment; error bars are the standard deviation of 3 independent trials.

We interpret the reduction in the fraction of cells with 2 GFP dots as evidence that the tetrameric Lac repressor is tethering the chromatids together, inhibiting the stretch of a correctly bi-oriented chromosome whose sister chromatids have attached to both poles. However, it is possible that the tetrameric Lac repressor generates fewer cells containing 2 GFP dots because it disrupts kinetochore assembly or slows error correction mechanisms in a way that the dimeric Lac repressor does not. If tetrameric Lac repressor disrupts kinetochores or inhibits error correction, a higher frequency of GFP-LacI_4_ bound chromosomes should be mis-segregated compared to GFP-LacI_2_ bound chromosomes. To test the segregation of GFP-LacI_2_ and GFP-LacI_4_ bound chromosomes, cells were arrested in anaphase using a temperature sensitive *cdc15-2* allele that inhibits mitotic exit [27]. Cells were synchronized in G1 with alpha factor, raised to the restrictive temperature, washed and released at the restrictive temperature to arrest cells in anaphase (Figure 3A). Cells were collected for scoring three hours after release from their G1 arrest, allowing cells to proceed to and arrest in anaphase as previously described [9], [27], [28]. Cells were stained with DAPI to confirm their arrest. Anaphase cells are large-budded and have DNA masses in each cell (Figure 3B); 99±1.5% of cells scored displayed this morphology. Correct segregation of the GFP-LacI bound chromosome was scored by the presence of one GFP dot in each mother and daughter cell, and mis-segregation was scored by one cell possessing both copies of the chromosome (two GFP dots in one cell) (Figure 3C). As a control, the segregation of GFP-labeled Chromosome III was also measured in cells with a conditional centromere. The *GAL1* promoter was placed upstream of *CEN3*; when cells are grown in glucose, the promoter is silent and the centromere functions normally (Figure 3D). When cells are grown in galactose, transcription initiated from the *GAL1* promoter disrupts centromere function and the chromosome is mis-segregated a high frequency [29]. Similar to previous studies using the conditional centromere [28], we found that 96±1% of cells grown in glucose correctly segregated the chromosome, but correct segregation occurred in only 41±6% of cells grown in galactose (Figure 3E). The presence of tetrameric Lac repressor did not disrupt chromosome segregation; both GFP-LacI_2_ and GFP-LacI_4_ bound chromosomes segregated correctly in 92±3% of cells. There was no statistical difference between cells grown in glucose, cells with GFP-LacI_2_, and cells with GFP-LacI_4_, but all were significantly different from cells grown in galactose (*p*≤0.003, Student's *t*-test). These results indicate that the presence of tetrameric Lac repressor does not disrupt kinetochore assembly or interfere with the correction of erroneous attachments, suggesting that the reduction in the fraction of metaphase-arrested cells with 2 GFP dots (Figure 2D) represents chromosomes that are correctly attached to opposite poles but cannot stretch apart due to the tethering effect of the tetrameric Lac repressor.

### Inhibiting chromatin stretch does not activate the spindle checkpoint

**Figure 4:**
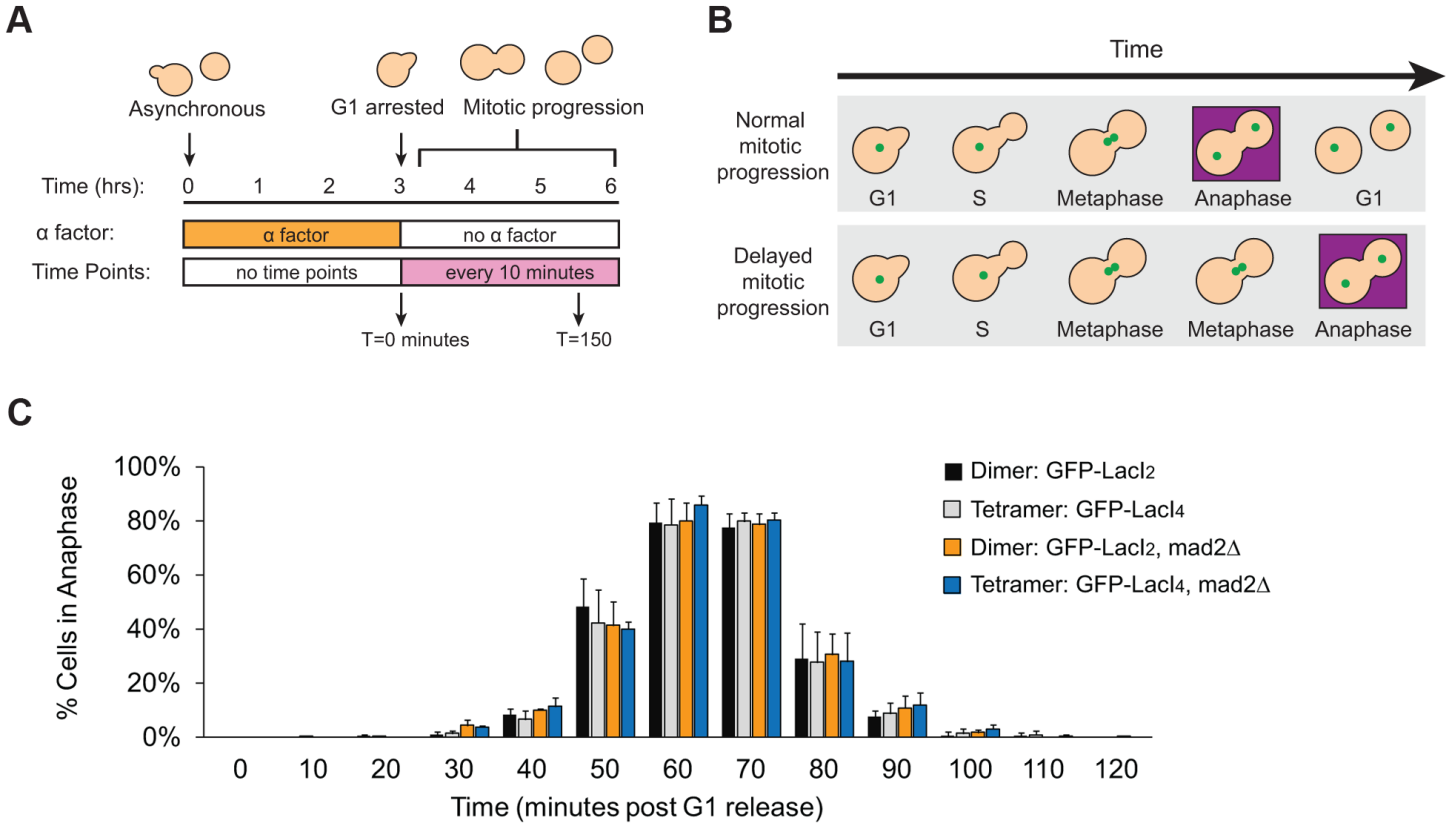
Inhibition of chromatin stretch does not delay mitotic progression. (**A**) To assay the progression of cells through mitosis, asynchronous populations were treated with alpha factor to arrest cells in G1. Cells were released from G1 and allowed to proceed synchronously through mitosis and into the next cell cycle. Samples were collected every 10 minutes and scored for mitotic progression (**B**) Cartoon of how the spindle checkpoint can arrest cells in metaphase, delaying anaphase. Anaphase (purple box) is scored in mitotic progression assays by the separation of GFP-labeled chromatids (green dots) into mother and daughter cells. (**C**) Cells expressing the tetrameric Lac repressor do not delay mitosis compared to control cells expressing the dimeric Lac repressor. Both strains peak in anaphase 60–70 minutes after release from G1 (no statistical difference between populations). The essential spindle checkpoint component Mad2 was deleted in cells expressing the dimeric or tetrameric Lac repressor, and all four strains move through mitosis on the same time scale. There was no statistical significance between any of the four strains at any one of the time points, suggesting that neither form of the Lac repressor delayed mitosis due to checkpoint activation. More than 100 cells were scored for anaphase for each strain in each experiment; error bars represent the standard deviation of at least 3 independent trials.

Does reduced inter-kinetochore separation produced by binding the tetrameric Lac repressor near the centromere activate the spindle checkpoint and thus delay the onset of anaphase? Cells were synchronized in G1 with alpha factor, washed and released to proceed through the cell cycle under conditions where they produce Cdc20, activate the APC, enter anaphase, and divide. Samples were taken every 10 minutes, fixed, and visualized to score mitotic progression (Figure 4A). Cells were scored for anaphase by the segregation of their GFP-labeled chromosome (Figure 4B). The separation of sister centromeres that indicates bi-orientation is always less than 1 µm, whereas the separation associated with anaphase is always greater than 2 µm, making it easy to rigorously distinguish the centromere separation associated with metaphase bi-orientation from the chromosome segregation of anaphase. The fraction of anaphase cells falls at the end of the experiment because cells divide, producing two daughter cells, each containing a single GFP dot. Control cells expressing GFP-LacI_2_ began to enter anaphase 40–50 minutes post-release from G1 and peaked with approximately 80% of cells in anaphase between 60 and 70 minutes. By 100 minutes, nearly every cell had exited mitosis (Figure 4C). Cells expressing GFP-LacI_4_ showed the same pattern of mitotic progression as control cells; they entered anaphase, reached a peak fraction of anaphase cells, and had fully exited mitosis at the same time as the GFP-LacI_2_ control (Figure 4C). At each time point, there was no statistically significant difference between control and tethered cells, suggesting that inhibition of chromatin stretch does not activate the spindle checkpoint. Since some of the cells that express GFP-LacI_4_ have not achieved the metaphase separation of sister centromeres after two hours in metaphase-arrested cells (Figure 2D), but cells that are allowed to pass through mitosis all complete anaphase within 90 minutes, we conclude that the failure to achieve metaphase centromere separation does not prevent entry into anaphase.

It is possible, however, that both GFP-LacI_2_ and GFP-LacI_4_ cells activated the spindle checkpoint and experienced mitotic delay. To rule out this possibility, we removed Mad2, an essential component of the spindle checkpoint, from both dimeric and tetrameric Lac repressor strains. All four strains (GFP-LacI_2_, GFP-LacI_4_, GFP-LacI_2_ *mad2Δ*, and GFP-LacI_4_ *mad2Δ*) moved through mitosis on the same time scale, with the peak of anaphase 60–70 minutes after release from G1 and with no statistically significant difference between any of the four strains (Figure 4C). These results show that neither the dimeric or tetrameric Lac repressor cause a mitotic delay by activating the spindle checkpoint.

**Figure 5:**
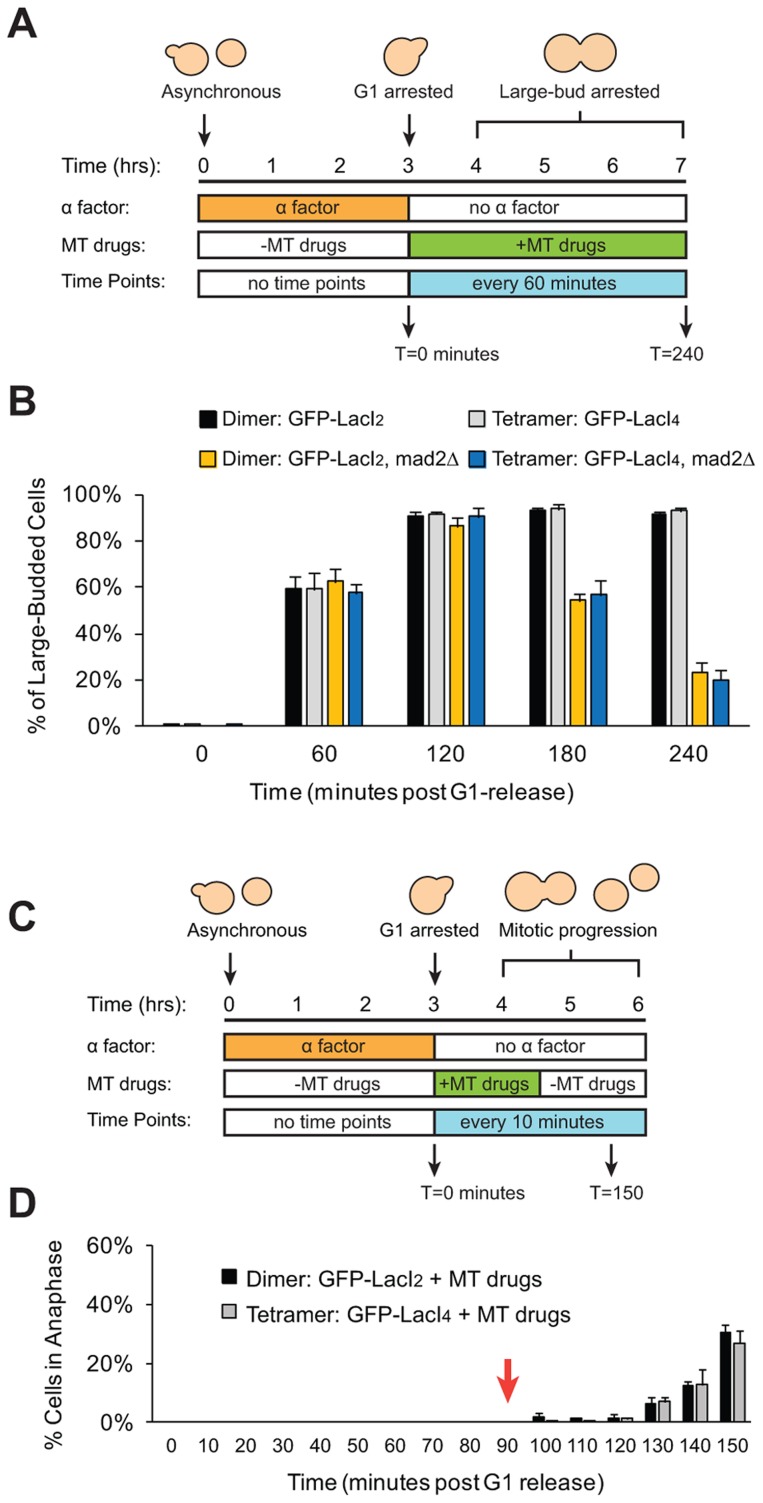
Cells with tethered centromeres can activate the spindle checkpoint. (**A**) To assay spindle checkpoint activation of cells in the presence of microtubule-depolymerizing drugs, cells were synchronized in G1 with alpha factor, and released into media containing microtubule-depolymerizing drugs (benomyl and nocodazole). Samples were collected every 60 minutes and scored for the large-budded phenotype indicative of checkpoint activation. (**B**) The ability of both control (dimeric) and tethered (tetrameric) cells to activate the spindle checkpoint was assayed by scoring the percent of cells arrested as large-budded cells, and compared to cells that do not possess a functional checkpoint and cannot arrest (*mad2Δ*). Cells expressing either the dimeric or tetrameric Lac repressor arrested in the drugs, indicating that the spindle checkpoint was functional in both strains. 200 cells scored for large-budded phenotype; error bars represent the standard deviation of 3 independent trials. (**C**) To assay the ability of cells to recover from spindle checkpoint activation induced by microtubule-depolymerizing drugs, cells were synchronized in G1 with alpha factor, released into media containing benomyl and nocodazole for 90 minutes then washed and transferred to media without drugs. Samples were collected every 10 minutes and scored for anaphase (GFP dots in both mother and daughter cells) (**D**) The ability of both control and tethered strains to recover from spindle checkpoint activation was assayed by transiently treating cells with microtubule-depolymerizing drugs. Both strains arrested in the presence of the drugs and did not enter anaphase until after the drugs were washed out at T = 90 minutes (red arrow). 100 cells were scored for anaphase for each strain in each experiment; error bars represent the standard deviation of at least 3 independent trials.

We wanted to eliminate the possibility that our manipulations had interfered with the checkpoint in either of two ways. The first is that introduction of the tethering components (Lac operator and either form of the Lac repressor) might disrupt the spindle checkpoint. The second is that tethering sister centromeres might activate the checkpoint and, as a result, strains containing the tetrameric Lac repressor could only be produced by selecting cells that have mutationally or epigenetically inactivated the checkpoint. To confirm that strains expressing either form of the Lac repressor can still activate the spindle checkpoint, cells were synchronized in G1 with alpha factor and released into the microtubule-depolymerizing drugs benomyl and nocodazole (Figure 5A). Treatment with these drugs activates the spindle checkpoint, preventing cells from going through mitosis and causing them to arrest as large-budded cells [5]. Approximately 90% of dimeric and tetrameric repressor-containing cells reached the large-budded stage 120 minutes after being released from G1 into microtubule poisons and remained arrested at this stage for the duration of the experiment (Figure 5B). Cells that lacked Mad2 (GFP-LacI_2_ *mad2Δ* and GFP-LacI_4_ *mad2Δ*) did not arrest; after peaking at a value of 90% at 120 minutes, the fraction of large-budded *mad2Δ* cells declined to 55% at 180 minutes and 20% at 240 minutes (*p*≤0.002 for all time points, Student's *t*-test), compared to the *MAD2* cells, 90% of which remained large-budded in the presence of the drugs. The difference between *mad2Δ* and *MAD2* cells was statistically significant at 180 and 240 minutes post-release (*p*≤0.005, Student's *t*-test). These results show that cells expressing the dimeric and tetrameric forms of the Lac repressor remain capable of activating the spindle checkpoint and arresting the cell cycle. To demonstrate that Lac repressor-containing cells can inactivate the spindle checkpoint and resume mitosis, cells expressing the dimeric or tetrameric Lac repressor were synchronized in G1 with alpha factor, released into benomyl and nocodazole. After 90 minutes of drug treatment, the cells were washed and transferred to drug-free media (Figure 5C). During drug treatment, no dimeric or tetrameric-expressing cells entered anaphase (0% anaphase cells through T = 90 minutes), but after drug wash-out (marked by red arrow) both dimeric and tetrameric cells recovered from the mitotic arrest and began entering anaphase (Figure 5D). By 150 minutes after their release from G1 arrest, approximately 30% of both GFP-LacI_2_ and GFP-LacI_4_ cells had entered anaphase. This result shows that both strains have functional spindle checkpoints that can be inactivated to allow cells to resume mitosis.

## Discussion

The spindle checkpoint ensures that all chromosomes are properly attached to the spindle; it monitors microtubule attachment to kinetochores and the tension generated when sister kinetochores attach to opposite spindle poles. We found that the binding of the Lac repressor to LacO arrays surrounding a budding yeast centromere holds sister kinetochores close together and we asked whether the checkpoint monitors tension within the kinetochore (L_1_ in Figure 1A) or responds to the distance between sister kinetochores (L_2_ in Figure 1A). Holding sister centromeres together did not activate the checkpoint, suggesting that the checkpoint senses tension by monitoring events within the kinetochore rather than responding to reduced distance between sister centromeres.

We compared the behavior of cells expressing tetrameric and dimeric forms of the Lac repressor to determine the effect of slowing the sister centromere separation associated with bi-orientation. By 60 minutes after their release from G1 into a metaphase arrest, the cells expressing GFP-LacI_2_ had reached a steady state, with half of them showing two GFP dots. This value is similar to previous observations [10],[11] and reflects oscillations in the distance between sister centromeres that can take their separation below the level detectable by light microscopy (“breathing”) [11], [30], [31]. The tetrameric repressor (GFP-LacI_4_) reduced the fraction of cells with visibly separated GFP dots (Figure 2D). Their percentage increased from 24% to 42% during the two hours the cells spent in metaphase, suggesting that spindle forces can gradually overcome the Lac repressor's tether, despite this tether being the tightest binding version of the Lac repressor [26]. We attribute the increase in the fraction of cells with one GFP dot to the tetrameric repressor holding chromatids together that are correctly attached to opposite poles (Figure 1C). To eliminate the possibility that tetrameric repressor increased the fraction of cells with one GFP dot cells by disrupting kinetochores or correction of erroneous attachments, we showed that the rate of chromosome mis-segregation is not increased in cells expressing the tetrameric repressor compared to control cells and those expressing the dimeric repressor (Figure 3E). Because the assay we used cannot reliably detect frequencies of chromosome mis-segregation below 5%, we cannot exclude the possibility that the presence of the tetrameric Lac repressor does not elevate the frequency of mitotic chromosome loss above the normal rate of 10^−5^/cell division. But we are confident that the long delay in separating sister chromatids in the cells expressing the tetrameric repressor is not due to their failure to attach to opposite spindle poles.

By repeating our experiment in cells that could enter anaphase, we showed that inhibiting sister centromere separation did not activate the spindle checkpoint to the point that delayed entry into anaphase. Cells expressing the dimeric and tetrameric forms of the Lac repressor progressed through mitosis indistinguishably: 60 minutes after their release from G1 arrest, most of the cells were in anaphase, even though there is a marked difference between the degree of inter-kinetochore stretch (23% of tetramer- versus 50% of dimer-expressing cells, Figure 2D) at this time in cells that have been arrested in metaphase. Observing the same kinetics of anaphase in cells expressing dimeric and tetrameric forms of the Lac repressor shows that inhibiting inter-kinetochore separation and thus the stretch of pericentric chromatin does not delay the cell cycle or the ability of microtubule-dependent forces to move kinetochores in anaphase (Figure 4C).

To eliminate the possibility that the dimeric and tetrameric versions of the Lac repressor were activating the spindle checkpoint, we tested the effect of removing Mad2, an essential component of the checkpoint. With either form of the repressor, the timing of mitosis is unchanged when the spindle checkpoint was deleted (Figure 4C), demonstrating that neither form activates the checkpoint. We also checked that our strains had a functional checkpoint. Cells expressing either form of the Lac repressor arrested as large-budded cells in response to microtubule depolymerization (Figure 5B), and the cells only entered anaphase once the microtubule-depolymerizing drugs were removed (Figure 5D).

Our results suggest that the spindle checkpoint does not monitor the distance between sister kinetochores. We cannot make this a rigorous conclusion because the tetrameric Lac repressor reduces inter-kinetochore separation rather than abolishing it. We can only detect that a higher fraction of centromere pairs are separated by a distance smaller than the resolution limit of our microscope, and despite the presence of the tetrameric Lac repressor, some cells still manage to produce visible, metaphase separation between sister centromeres. Nevertheless, we might expect that some of the cells that express the tetrameric repressor have their sister centromeres close enough together to activate the spindle checkpoint and thus that some of the cells in this strain would enter anaphase more slowly than the control strain expressing the dimeric repressor. We see no such effect, leading us to argue that the checkpoint does not monitor inter-kinetochore distance. We assayed for spindle checkpoint activation by mitotic progression; cells that had activated the checkpoint should be delayed in entering anaphase [5], [6]. The sensitivity of our assay would reveal if cells with tethered kinetochores are delayed in metaphase by 10 minutes or more, but we cannot rule out transient checkpoint activation on a shorter time scale. Unfortunately, no other method for assaying spindle checkpoint activation would provide greater resolution for activation caused by a single chromosome in budding yeast. Unlike higher eukaryotes, methods such as visualizing Mad1 or Mad2 bound to individual kinetochores are not feasible in budding yeast because kinetochores are too clustered to distinguish individual kinetochores, and localization of these checkpoint proteins to kinetochores has only been demonstrated in response to global spindle defects [32], [33]. Nevertheless, because it takes much longer to overcome the tether in metaphase arrested cells (Fig. 2D), than it takes the same cells to proceed through an unrestrained mitosis (Fig. 4C), we argue that the presence of the tether does not substantially activate the spindle checkpoint.

### Intra-kinetochore stretch models for tension-sensation

If the checkpoint does not monitor events between sister centromeres, it must respond to changes within the kinetochore. Maresca and Salmon [12] showed that treating *Drosophila melanogaster* tissue culture cells with taxol reduces inter-kinetochore but not intra-kinetochore stretch and does not activate the spindle checkpoint. Uchida et al. [13] showed that treating HeLa cells with low nocodazole concentrations reduces intra-kinetochore but not inter-kinetochore stretch and does activate the checkpoint. Our studies agree with the conclusion that the checkpoint responds to events within kinetochores rather than between them: we find that inhibiting chromatin stretch does not activate the checkpoint, and our approach avoids the potential side effects of altering microtubule dynamics with drugs, and isolates chromatin stretch from other effects on spindle structure and dynamics.

Kinetochores can elongate under tension [12]–[14]. In *Drosophila* S2 cells, unattached kinetochores measure 65±31 nm from the inner centromere protein, CENP-A, to the outer kinetochore protein, Ndc80. When attached and bi-oriented, this distance increases by an average of 37 nm [12]. Kinetochores could elongate by two mechanisms: altering their composition [34] or changing the conformations and contacts of individual proteins. Studies using immuno-electron and fluorescent microscopy showed that inner kinetochore proteins CENP-A, -C, and -R deform under tension, and CENP-T elongates, separating its N- and C-termini [35]. The outer domains of the microtubule-binding Ndc80 complex has also been shown to move 15 nm further away from the inner kinetochore upon bi-orientation [14], perhaps by straightening of a long coiled-coil domain broken by a flexible, elbow-like hinge [36].

Two different mechanisms have been proposed for the link between kinetochore elongation and the activity of Ipl1: relaxing the kinetochore activates Ipl1, or it allows an already activated kinase better access to its substrates. In budding yeast, Bir1 and Sli15 (Survivin and INCENP in higher eukaryotes), members of the chromosomal passenger complex that localize and activate Ipl1, help link centromeres and microtubules [37], [38]. Studies on *SLI15* and *BIR1* mutants have led to the proposal that these proteins activate Ipl1 on relaxed kinetochores [37]. Recently, it has been shown that Sli15's ability to cluster Ipl1 together rather than its ability to localize the kinase to the centromere may be sufficient for distinguishing between correct and incorrect attachments [39]. There is also evidence supporting a constitutively active kinase that is separated from its substrates when the kinetochore is stretched: the phosphorylation of an Ipl1/Aurora B target depends on its distance from the kinase, located in the inner kinetochore, and repositioning the kinase closer to the outer kinetochore destabilizes microtubule attachments and activates the checkpoint [40].

Our results in yeast corroborate other work arguing that the spindle checkpoint measures the effects of tension within kinetochores. Monitoring the kinetochore means that the checkpoint would not activate in response to the observed variations in the distance between sister chromatids, but would detect mono-oriented chromosomes. Preventing false alarms from a tensiometer at the kinetochore would requires it to have one of two properties to keep the checkpoint from activating as the distance between sister centromeres fluctuates: 1) the extensible element within the kinetochore would have to have a lower spring constant than the linkage between the centromeres to make sure the tensiometer remained stretched, or 2) the conformational change that activated the checkpoint would have to be slower than the variations in the overall force separating the sister centromeres. Distinguishing between these possibilities will require further investigation of kinetochore dynamics and biochemistry.

## Materials and Methods

### Yeast strains and culturing

Strains used in this study are listed in Table 1; all were constructed in W303 (*ade2-1 his3-11,15 leu2-3,112 trp1-1 ura3-1 can1-100*) using standard genetic techniques. Lactose operator arrays containing 256 repeats of the operator were integrated either upstream of the centromere or on either side of the centromere on Chromosome III. Both arrays were integrated approximately 1500 bp from the centromere. Dimeric control strains contained a C-terminal truncation mutant of the Lac repressor (LacI_2_) that cannot cross-link two arrays; experimental cells contained the wild-type version of the Lac repressor capable of tetramerizing and cross-linking two arrays (LacI_4_) [24]. Both versions of the repressor were placed under the *HIS3* promoter and were fused via their N-terminus to monomeric yeast optimized GFP. Cells were either grown in Synthetic Complete media (2% glucose) lacking histidine (SC-HIS) or Synthetic Complete media (2% glucose) lacking histidine and methionine (SC-HIS-MET) at 30°C to promote expression of the Lac repressor under the *HIS3* promoter. YPD containing 1-(butylcarbamoyl)-2-benzimidazolecarbamate (benomyl) and nocodazole was prepared by heating YPD to 65°C and adding dimethyl sulfoxide (DMSO) 10 mg/ml stocks of benomyl drop-wise to a final concentration of 30 µg/ml; media was cooled to 37°C for drop-wise addition of DMSO 10 mg/ml stock of nocodazole to a final concentration of 30 µg/ml. All drugs and chemicals were purchased from Sigma Aldrich.

**Table 1:** Strains used in this study.

Strain Name	Genotype
**yNJN175**	*MATa, P_HIS3_-GFP-LacI_2_::HIS3, LacO(256)::LEU2::1.5kb upstream CEN3, LacO(256)::URA3::1.5kb downstream CEN3*
**yNJN176**	*MATa, P_HIS3_-GFP-LacI_4_::HIS3, LacO(256)::LEU2::1.5kb upstream CEN3, LacO(256)::URA3::1.3kb downstream CEN3*
**yNJN210**	*MATa, P_HIS3_-GFP-LacI_2_::HIS3, LacO(256)::LEU2::1.5kb upstream CEN3, LacO(256)::URA3::1.5kb downstream CEN3, P_Met_-HA3-Cdc20::TRP1*
**yNJN211**	*MATa, P_HIS3_-GFP-LacI_4_::HIS3, LacO(256)::LEU2::1.5kb upstream CEN3, LacO(256)::URA3::1.5kb downstream CEN3, P_Met_-HA3-Cdc20::TRP1*
**yNJN489**	*MATa, P_HIS3_-GFP-LacI_2_::HIS3, LacO(256)::LEU2::1.5kb upstream CEN3, LacO(256)::URA3::1.5kb downstream CEN3 mad2Δ*
**yNJN490**	*MATa, P_HIS3_-GFP-LacI_4_::HIS3, LacO(256)::LEU2::1.5kb upstream CEN3, LacO(256)::URA3::1.5kb downstream CEN3 mad2Δ*
**yNJN503**	*MATa, P_HIS3_-GFP-LacI_2_::HIS3, LacO(256)::LEU2::1.5kb upstream CEN3, P_Met_-HA3-Cdc20::TRP1*
**yNJN504**	*MATa, P_HIS3_-GFP-LacI_4_::HIS3, LacO(256)::LEU2::1.5kb upstream CEN3, P_Met_-HA3-Cdc20::TRP1*
**yNJN510**	*MATa, P_HIS3_-GFP-LacI_2_::HIS3, LacO(256)::LEU2::1.5kb upstream CEN3, LacO(256)::URA3::1.5kb downstream CEN3, cdc15-2*
**yNJN511**	*MATa, P_HIS3_-GFP-LacI_4_::HIS,3 LacO(256)::LEU2::1.5kb upstream CEN3, LacO(256)::URA3::1.3kb downstream CEN3, cdc15-2*
**SLY849**	*MATa, P_HIS3_-GFP-LacI_2_::HIS3, P_HIS3_-GFP-LacI_2_::ADE2, LacO(256)::LEU2, P_GAL1_-CEN3::TRP1, URA3::1.6kb downsteam CEN3, cdc15-2*

All strains are derivatives of *Saccharomyces cerevisiae* W303 with the following auxotrophic genotypes: *ade2-1 can1-100 his3-11,15 leu2-3,112 trp1-1 ura3-1*.

### Chromatin stretch assay

Strains were grown in SC-HIS-MET at 30°C and maintained in log phase for 24 hours before the experiment. Log phase cells (∼5×10^6^ cells/ml) were arrested in G1 with 10 µg/ml alpha factor (Bio-Synthesis) for 3 hours. After confirmation of arrest by light microscopy, cells were washed three times with YPD to remove alpha factor and released into SC-HIS media containing methionine (250 µg/ml). Media lacking methionine allows cells to grow, but media containing methionine inhibits expression of Cdc20 from the *MET* promoter and induces metaphase arrest. Cells were grown at 30°C for 3 hours, and samples were collected every 30 minutes (see Figure 2A). Samples were fixed with formalin (see below) and stored at 4°C for imaging. Using fluorescence microscopy to visualize GFP-tagged chromatids, samples were scored for the presence of one or two GFP dots; two dots indicates stretched chromatids.

### Chromosome segregation assay

Strains were grown in SC-HIS plus 2% raffinose at 23°C and maintained in log phase for 24 hours before the experiment. Log phase cells (∼5×10^6^ cells/ml) were arrested in G1 with 10 µg/ml alpha factor (Bio-Synthesis) for 3 hours at 23°C. Cells were transferred to either SC -HIS+2% galactose+10 µg/ml alpha factor to induce the *GAL1* promoter or to SC-HIS+2% glucose+10 µg/ml alpha factor to repress the promoter, and G1 synchronization continued an additional hour at the restrictive temperature (37°C). After confirming the arrest by light microscopy, cells were then washed three times in YEP, and incubated for a further three hours in either SC-HIS+2% glucose or 2% galactose at 37°C. Under these conditions, cells proceed through the cell cycle and arrest at anaphase, as large-budded cells because of the *cdc15* mutation (see Figure 3A). Samples were sonicated, fixed with formalin (see below), and stored at 4°C for imaging. Cells were scored for chromosome segregation based the position of the two chromatid copies of GFP-labeled chromosome III. Correct chromosome segregation produces one copy of the chromosome (one GFP dot) in both the mother and daughter cells, whereas incorrect chromosome segregation leads to two GFP dots in a single cell. Anaphase arrest was confirmed by staining fixed cells with ProLong Gold antifade reagent with DAPI (Life Technologies); 100 cells were scored in three independent trials for DNA masses in both mother and daughter cells.

### Mitotic progression assay

Strains were grown in SC-HIS at 30°C and maintained in log phase for 24 hours before the experiment. Log phase cells (∼5×10^6^ cells/ml) were arrested in G1 with 10 µg/ml alpha factor (Bio-Synthesis) for 3 hours. After confirmation of arrest by light microscopy, cells were washed three times with YPD to remove alpha factor and released into SC-HIS media. Cells were grown at 30°C for 3 hours, and samples were collected every 10 minutes (see Figure 4A). Samples were sonicated, fixed with formalin (see below), and stored at 4°C for imaging. After 60 minutes, 10 µg/ml alpha factor was added to prevent additional entry into a second mitosis during the experiment. Samples were scored for mitotic progression by cell morphology and position of GFP-tagged chromatids. Anaphase was scored as large-budded cells with GFP-tagged chromatids separated into mother and daughter cells.

### Spindle checkpoint activation assay

Strains were grown in SC-HIS at 30°C and maintained in log phase for 24 hours before the experiment. Log phase cells (∼5×10^6^ cells/ml) were arrested in G1 with 10 µg/ml alpha factor (Bio-Synthesis) for 3 hours. After confirming the arrest by light microscopy, cells were washed three times with YPD to remove alpha factor and released into YPD containing 30 µg/mL 1-(butylcarbamoyl)-2-benzimidazolecarbamate (benomyl) and 30 µg/mL nocodazole prepared as described above. In Figure 5B, cells were grown in the drugs at 30°C for 4 hours with samples collected every 60 minutes and scored for the percentage of large-budded cells. In Figure 5D, cells were grown in the drugs at 30°C for 90 minutes then washed three times with YPD and released into drug-free YPD for an additional 60 minutes of growth at 30°C. Samples were taken every 10 minutes post-release from G1, fixed with formalin (see below) and scored for anaphase, identified as large-budded cells with GFP-tagged chromatids separated into mother and daughter cells.

### Sample fixation and imaging by fluorescent microscopy

Samples for imaging were fixed with 10% formalin added directly to growth media containing cells (final concentration of 1%), incubated for 10 minutes at room temperature, washed with 0.1M KH_2_PO_4_ pH 8.5, washed with 1.2M Sorbitol+0.1M KH_2_PO_4_ pH 8.5, resuspended in 1.2M Sorbitol+0.1M KH_2_PO_4_ pH 8.5, and stored at 4°C. Images were acquired at room temperature (25°C) using a Nikon Eclipse Ti-E inverted microscope with a 60× Plan Apo VC, 1.4 NA oil objective lens with a Photometrics CoolSNAP HQ camera (Roper Scientific). Metamorph 7.7 (Molecular Devices) was used to acquire images. Fixed samples were imaged in 1.2M Sorbitol+0.1M KH_2_PO_4_ pH 8.5 buffer on Concanavalin A-coated coverslips (VWR) adhered to glass slides (Corning). Exposure times were 10 ms for differential interference contrast and 300 ms for fluorescence.
